# Study into the Mechanical Properties of a New Aeronautic-Grade Epoxy-Based Carbon-Fiber-Reinforced Vitrimer

**DOI:** 10.3390/polym14061223

**Published:** 2022-03-17

**Authors:** Cristian Builes Cárdenas, Vincent Gayraud, Maria Eugenia Rodriguez, Josep Costa, Asier M. Salaberria, Alaitz Ruiz de Luzuriaga, Nerea Markaide, Priya Dasan Keeryadath, Diego Calderón Zapatería

**Affiliations:** 1 Composites Unit, Eurecat—Technological Center of Catalonia, 08290 Cerdanyola del Vallès, Spain; vincent.gayraud@eurecat.org; 2 Analysis and Advanced Materials for Structural Design (AMADE), Polytechnic School, Campus Montilivi, University of Girona, 17071 Girona, Spain; josep.costa@udg.edu; 3 CIDETEC, Basque Research and Technology Alliance (BRTA), Paseo Miramón, 196, 20014 Donostia-San Sebastián, Spain; amartinez@cidetec.es (A.M.S.); aruiz@cidetec.es (A.R.d.L.); nmarkaide@cidetec.es (N.M.); 4 ÉireComposites Teo, An Choill Rua, H91 Y923 Inverin, County Galway, Ireland; info@eirecomposites.com; 5 IDEC, Engineering Composites Advanced Solution, C/Albert Einstein 29, 01510 Miñano Menor, Spain; d.calderon@idec.aero

**Keywords:** vitrimers, carbon fiber composites, aeronautical industry, mechanical properties

## Abstract

The current drive for sustainability demands recyclable matrices for composite materials. Vitrimers combine thermoset properties with reprocessability, but their mechanical performance in highly loaded applications, for instance, composites for aeronautics, is still to be demonstrated. This work presents the complete mechanical characterization of a new vitrimer reinforced with carbon fiber. This vitrimer formulation consists of functional epoxy groups and a new dynamic disulfide crosslinks-based hardener. The testing campaign for the vitrimer composites encompassed tension, compression, interlaminar shear strength (ILSS), in-plane shear (IPS), open-hole tension (OHT) and compression (OHC), filled-hole compression (FHC) and interlaminar fracture toughness tests under mode I and II. Test conditions included room temperature and high temperature of 70 °C and 120 °C, respectively, after moisture saturation. Tension and flexural tests also were applied on the neat vitrimer resin. The results compared well with those obtained for current aeronautic materials manufactured by Resin Transfer Molding (RTM). The lower values observed in compression and ILSS derived from the thermoplastic veils included as a toughening material. This work demonstrates that the vitrimer formulation presented meets the requirements of current matrices for aeronautic-grade carbon-reinforced composites.

## 1. Introduction

Thermoset fiber-reinforced composites have been widely used in numerous structural applications, particularly in the aerospace, automotive and wind power industries. Epoxies have excellent thermal and dimensional stability, good mechanical strength, creep and chemical resistance and good electrical isolation thanks to their permanent covalent cross-linked networks. That said, thermosets have long curing times, thus limiting their production to low-medium volume series, for which there is an increasing demand. In addition, thermosets have poor or complex reparability, are non-recyclable and cannot be reshaped after being cured, therefore generating a great deal of waste when components reach the end of their lifespans. The current and most common disposal solutions are pyrolysis and land filling, both of which imply serious environmental and economic issues [1,2,3,4,5,6,7].

Back in 2011, Leibler et al. [8] presented a new kind of polymer with outstanding properties called covalent adaptative networks (CANs), dynamers or vitrimers that combine the performance of traditional thermosets with the versatility of thermoplastics due to characteristics such as processability, weldability, self-healing capacity and recyclability. Vitrimers, having associative dynamic networks, behave like traditional thermoset resins at service temperatures due to their frozen network topology. However, under certain stimuli, such as heat or light, they are able to reorganize their networks while maintaining a constant number of chemical bonds. The dissociative formulations reduce network crosslinking density, thus diminishing polymer dimensional stability and viscosity during reprocessing. Some vitrimer formulations need the addition of external catalysts to create the bond exchange reactions, as the material is heated above the vitrimeric transition temperature, therefore presenting issues with vitrimer stability, manipulation and mechanical response. Some studies present new formulations of catalyst-free vitrimers that can be processed with commercially available precursors, maintaining their properties during reprocessing and are more easily implemented on an industrial scale [2,9,10,11,12,13,14,15,16,17,18,19].

Vitrimers present new possibilities for fiber-reinforced polymers such as post-curing reprocessing without losing performance, welding composite layers to form new monolithic and multi-layered materials or allowing composites to be welded to generate structural joints between parts. Additionally, the fibers and the matrix can be separated for reuse in other applications. Furthermore, vitrimers allow for the implementation of “enduring” prepregs that could be fully cured and still enable the processing of multi-layered laminates, thus avoiding the need for refrigerated storage and the multiple consumable materials for prepreg protection, in addition to offering a longer shelf life. Vitrimers offer the potential to create reprocessable and reparable thermoset composites, something which currently is a slow and expensive process requiring highly qualified personnel [9,11,20,21,22,23,24].

A pioneering publication on fiber-reinforced vitrimers by Ruiz de Luzuriaga, A. et al. [9] presented a glass-fiber-reinforced vitrimer based on diglycidyl ether of bisphenol A (DGEBA) epoxy monomer and a 4-aminophenyl disulfide dynamic hardener. This is a catalyst-free formulation that presents fast stress relaxation at high temperatures. Compared to a traditional DGEBA epoxy with diethyltoluenediamine (DETDA, 2) hardener, the disulfide vitrimer showed comparable values of glass transition temperature (T_g_), storage modulus (*E*′) and thermal stability, albeit with a slightly lower degradation temperature (T_d_), possibly due to the disulfide species that are less energetically stable than other species in the formulation. Both the vitrimer and the thermoset showed comparable traction strength, suggesting that the use of dynamic formulations on composites will not affect their mechanical performance. The authors demonstrated the possibility of reshaping a cured composite sample manufactured by RTM and thermoforming it in a hot press. The reparability of the vitrimer formulation was illustrated by inducing delamination during an ILSS test and healing it by means of heating and pressure.

Over the past years, several vitrimer composites with diverse synthetic or natural monomers and dynamic formulations have been applied in continuous glass or carbon fiber reinforcements, showing promising mechanical properties compared to their thermoset counterparts [6,10,19,20,23,25,26,27,28,29,30,31,32,33,34,35,36,37]. Some vitrimer formulations have attractive properties such as a high tensile strength, interlaminar shear strength or glass transition temperatures [6,23]. However, there is still a gap between their performance and current aerospace-grade structural thermoset composites (as displayed in Table 1), with no reported aerospace-capable vitrimer formulations yet. No detailed mechanical studies on vitrimer-based composites relevant to the aviation industry are available for use in evaluating the possible implementation of high-performance fiber-reinforced vitrimers in real structures.

The EU H2020 project AIRPOXY (thermoformAble, repaIrable and bondable smaRt ePOXY-based composites for aero structures) [41,42] was funded with the aim of introducing vitrimer advantages into the aeronautic industry to reduce production and maintenance costs of aeronautic composite parts. Vitrimer-based composites offer new properties such as reprocessability, reparability and recyclability, while still maintaining high-performance. 

This work is developed as part of the AIRPOXY project, and its objective is to present a full mechanical characterization of a new carbon-fiber-reinforced vitrimer based on disulfide exchange bonds, designed to compete in performance with current and widely used aerospace-grade thermoset epoxy resins. In this paper, we focus on composite properties; details of the vitrimer formulation and its properties will be published in separate works under the Airpoxy project. Two reinforcement configurations were selected and evaluated under different mechanical tests at room temperature and in humidity and high-temperature conditions to obtain a full spectrum of this vitrimer formulation’s mechanical performance. We present a description of the mechanical tests selected, along with their outcomes, materials and manufacturing processes. Following that, we present a discussion on the results and draw some conclusions. 

## 2. Experimental

### 2.1. Materials

We used a new formulation of an aeronautical-grade epoxy-based vitrimer (referred to from here on as AIR-3R) that has low viscosity at processing temperatures so that it can be processed by infusion and RTM processes. It consists of a catalyst-free formulation composed of commercially available functional epoxy groups and a new hardener with dynamic crosslinks based on aromatic disulfide species, also commercially available. This is a patented formulation, created, characterized and provided by Cidetec (Donostia—San Sebastián, Spain) [43,44,45].

**Figure 1:**
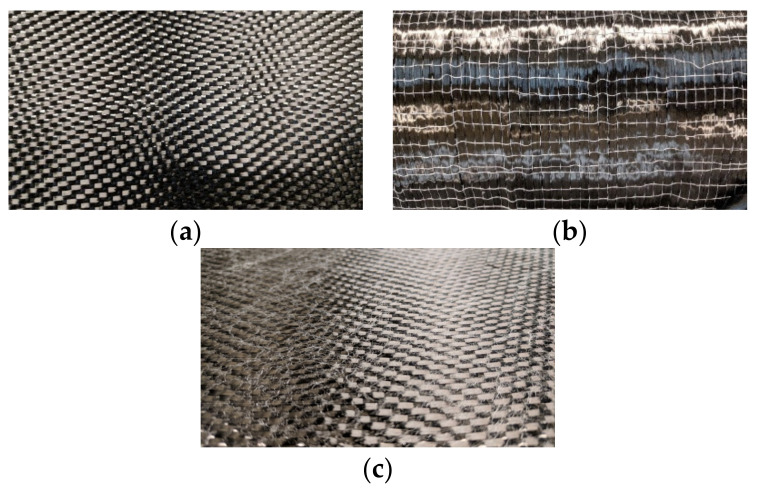
Fabric reinforcement structures used in this study. (**a**) 5-Harness satin weave. (**b**) UD fabric. (**c**) Detail of PA binder veil.

Two different carbon fiber reinforcements provided by Chomarat (Le Cheylard, France) were used in this study. One was C-WEAVE™ 280SA5 (Figure 1a), which is 5-harness satin (5HS) woven fabric with T800HB 6K (Chomarat) intermediate modulus fibers and an aerial weight of 280 g/m^2^ [46]. The other reinforcement was C-PLY™ SP U268S5 (Chomarat) (Figure 1b), which is a unidirectional (UD) fabric with T800H 24K intermediate modulus fibers and an aerial weight of 284 g/m^2^. Both reinforcements were manufactured with a custom-made polyamide (PA) stabilization veil (Chomarat) with an aerial weight of 8 g/m^2^, intended to improve the interlaminar toughness of the final vitrimer composites. (Figure 1c). The fiber volume content (FVC) for both vitrimer composites and thermoset baseline reference was set to 58%, considering the fabrics characteristics, samples thickness and number of fabric plies [47,48].

To characterize the 5HS fabric, we tested cross-ply configurations (CP), while for the UD reinforcement, 0° and 90° configurations were used. Laminate tests were performed over a quasi-isotropic configuration (Table 2).

Materials were tested as received at room temperature (RT) or preconditioned until equilibrium at 70 °C and 85% relative humidity (RH) and then tested at two different temperatures, 70 °C (HW70) and 120 °C (HW120).

Open-hole tension and compression and filled-hole Compression tests were carried out at RT and HW70 conditions. Interlaminar fracture toughness tests were conducted at RT.

### 2.2. Manufacturing Procedure

Panels were prepared by RTM using three-part metallic molds: upper and lower mold parts with an intermediate spacer, which gives the samples their final thickness required for the mechanical tests. Cavity dimensions were 505 mm × 605 mm for a steel mold and 380 mm × 220 mm for an aluminum mold. One of the two molds was selected depending on the test preparation, specimen dimensions or availability. Both were heated with an oil-based electric heater Tool Temp TT-380 (Tool Temp. Rubí, Spain) of 32 kW capacity. Injection was carried out on a Coexpair Injector 5000cc RTM machine (Coexpair. Namur, Belgium).

**Figure 2:**
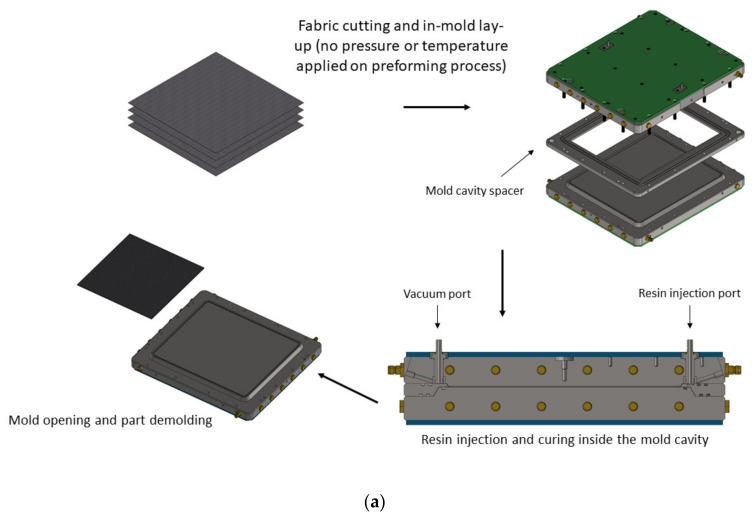

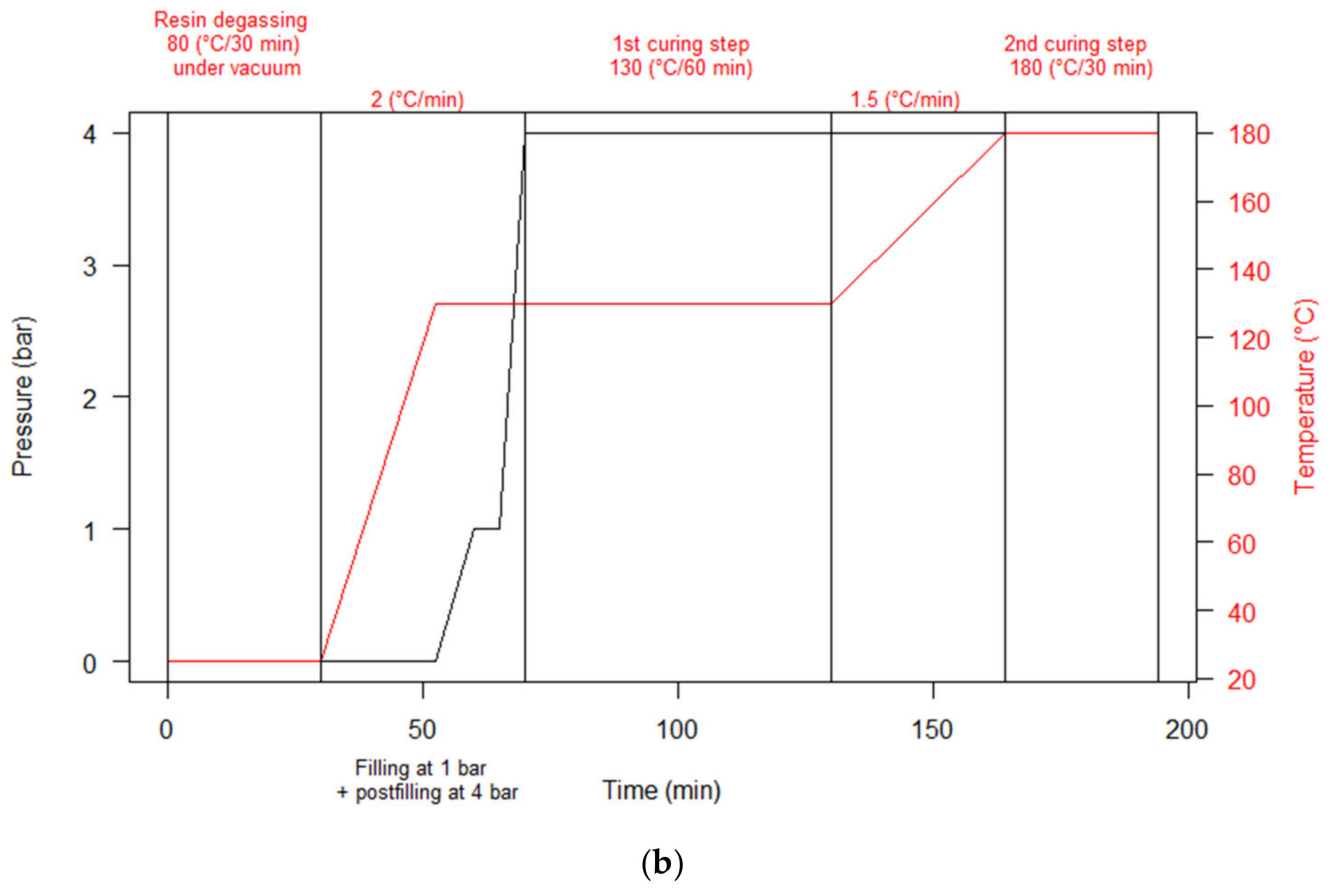
Detail of manufacturing procedure. (**a**) Process steps for composite manufacture. (**b**) Process conditions for injection and curing.

Dry fabrics were cut and manually placed inside the mold cavity with the specific reinforcement type and stacking sequence for each test. No pressure or temperature change was applied to the laminate during the preforming process, as displayed in Figure 2a. Injection was carried out at a constant rate of 50 g/min, maintaining a maximum pressure of 1 bar. When the mold cavity was full, a post-filling step was applied, thus increasing the cavity pressure up to 4 bars to ensure full preform impregnation, constant fiber volumetric fraction and less void content [49,50]. Heating slopes and curing steps are described in Figure 2b.

**Figure 3:**
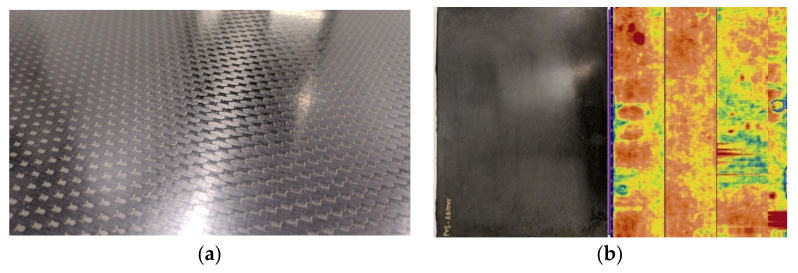
Preparation of AIR-3R test samples. (**a**) Visual inspection of the carbon fiber sheet manufactured inside the RTM molds. (**b**) Ultrasonic C-Scan inspection of the manufactured carbon fiber sheets prior to samples cutting.

Samples were validated prior to machining through visual inspection and C-scans (Figure 3a,b), and cutting was done using a waterjet.

### 2.3. Tests and Equipments

Neat resin tensile tests were performed following the ISO 527-2:2012 standard [51] on five dumbbell-shaped specimens (type 1B) of 150 mm in total length, 20 mm in total width and a thickness of 4 mm. The testing area had a length of 50 mm and a width of 10 mm. Test was carried out at a speed of 1 mm/min.

Neat resin flexural tests were performed following the ISO 178:2019 standard [52] in a three-point test configuration. The specimen dimensions were 80 mm length, 10 mm width and 4 mm thickness. Five specimens were tested at a speed rate of 2 mm/min.

Composites tensile tests were performed following the ISO 527-4:1997 standard [53] on specimens of 250 mm × 25 mm with 2 mm thickness (tabbed type 3) for the 5HS fabric and 250 mm × 15 mm and 1 mm thickness for the UD. Five specimens were tested for each reinforcement. Tests were carried out at a speed of 2 mm/min. A biaxial extensometer was applied to obtain strain measurements.

Neat resin tensile and flexural test and composite tensile tests were carried out on an Instron 5985 universal testing machine (Barcelona, Spain).

Compression tests were performed following the ISO 14126:1999 standard for the 5HS fabric [54] at the speed of 0.5 mm/min. Ten 110 mm × 10 mm specimens with 2 mm thickness (tabbed type A) were tested. The UD tests were performed following the ASTM D3410/B standard [55] with specimen dimensions of 150 mm × 10 mm × 3 mm. Strain gages were applied on both sides of the specimens. An anti-buckling device was used following the requirements from the ASTM standard (ITTRI test fixture).

Interlaminar shear strength tests were performed following the ISO 14130:1997 standard [56] at a loading rate of 1 mm/min. Specimen dimensions were 20 mm × 10 mm × 2 mm thickness.

In-plane shear tests were performed following the ISO 14129:1997 standard [57] with a loading rate of 2 mm/min. Specimen dimensions were 250 mm × 25 mm × 2 mm thickness (tabbed specimens). Deformations were measured using a biaxial extensometer.

Open-hole tension and compression tests were performed following ASTM standards D5766M [58] and ASTMD6484M [59], respectively. Specimens were 3 mm thick, 300 mm long and 36 mm wide. The hole diameter was 6 mm. Tests were carried out at speeds of 2 mm/min (OHT) and 1 mm/min (OHC).

Filled-hole compression tests were performed following the ASTM D6742M standard [60]. Specimen dimensions were 300 mm × 36 mm with 3 mm thickness and 6 mm hole diameter. The specimen was mounted with a titanium protruding-head HI-LOK DAN7-8-3 fuse pin (IDEC, Vitoria, Spain). Tests were carried out on five specimens at a speed of 1 mm/min. A specific support fixture was used to prevent the coupons buckling in the OHC and FHC tests.

Compression, ILSS, IPS, OHT, OHC and FHC tests were carried out on an MTS series 332.31 dynamic testing machine (SEM Engineering. Barcelona, Spain). Two different load cells were used: MTS 661.20F-02 50 kN load cell for the ILSS test and MTS 661.22D-01 250 kN for compression, IPS, OHT, OHC and FHC tests.

Mode I fracture toughness (G_IC_) tests were performed following the EN 6033:2015 standard [61]. Sample dimensions were 250 mm × 25 mm × 3.2 mm thickness. Inside the specimens, 0.01 mm-thick PTFE release film was used to create a crack in the laminate. Tests were carried out on a ZwickRoell 3 testing machine at a speed of 10 mm/min. Test was applied in the interlaminar configuration.

Mode II fracture toughness (G_IIC_) tests were performed following standard EN 6034:2015 [62]. The sample length was 115 mm long, 25 mm wide and 3 mm thick. Tests were carried out on a ZwickRoell 3 testing machine (Leominster, United Kingdom) at a speed of 1 mm/min. Test was applied in the interlaminar configuration.

The glass transition temperature was measured following the ISO 11357-2:2013 standard [63] through differential scanning calorimetry test (DSC). Samples were heated until 230 °C at a rate of 10 °C/min. Two heating steps were applied, and tests were carried out on a DSC Q20 TA Instruments machine (Cerdanyola del Vallès, Spain).

### 2.4. Baseline Properties

A set of baseline material properties relevant for aeronautical applications were defined in order to compare the AIR-3R resin behavior as a matrix for fiber-reinforced composites [47]. The objective of this vitrimer composite is to be mechanically comparable to and compete with the most widely used thermoset resin systems in the RTM process in the aeronautical industry, such as the HexFlow^®^ RTM6 from Hexcel (Madrid, Spain) [64]. Baseline data was scaled up to 10% to account for the fact that the reference composite properties have been obtained with a standard modulus fiber, whereas in this work, we use an intermediate modulus fiber (around 20% difference in the respective tensile modulus [65,66,67,68]). All conventional thermoset values were collected with samples manufactured by RTM using the RTM6 resin (referred to as STD-AR from here on). Baseline values are summarized in Table 2.

## 3. Results and Discussion

### 3.1. T_g_

The T_g_ of the AIR-3R vitrimer obtained by DSC was 170 °C ± 3 °C, which is equal to the baseline requirement. The DSC showed that the current curing process led to a degree of complete curing. AIR-3R had a lower T_g_ than the standard aeronautic thermoset references (Table 1) but was still much higher than most of the vitrimer formulations reported so far. The VA vitrimer from Wang, S. et al. [23] shows almost the same T_g_ value due to the high rigidity of the Schiff-based structure, as the cross-linking density was proved to be low, and the vitrimer formulation made by Yuan, Y. et al. [6] exhibited a higher T_g_, being more equivalent to the aeronautic thermoset references, also due to the strong network structure, composed in this case by C-N covalent bonds.

We measured the water uptake of the AIR-3R vitrimer under 70 °C/85% RH, with a mass gain of 2.3% after 30 days until moisture equilibrium. The measured T_g_ after this was 155 °C, having a reduction of 8.8% of the pristine value, validating related findings on the effects of moisture on the glass transition temperature of epoxies [69,70]. The T_g_ value of the aged AIR-3R vitrimer is still above the highest temperature and humidity condition, meaning that it should not affect its final performance under these conditions.

### 3.2. Neat Vitrimer Tension

**Figure 4:**
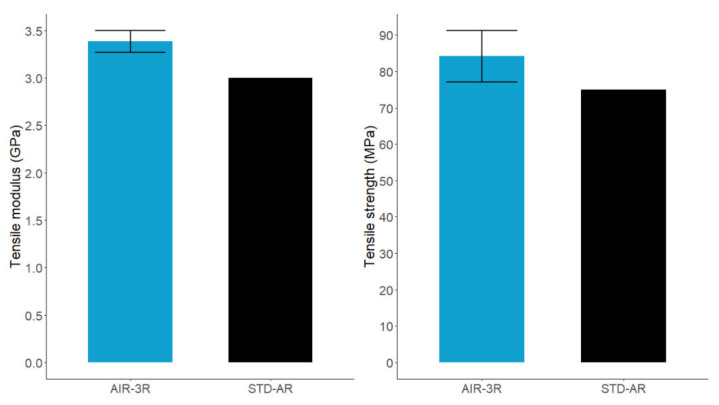
Tensile modulus (**left**) and strength (**right**) for the neat vitrimer and baseline reference.

The AIR-3R neat vitrimer resin presented better tensile modulus and tensile strength (Figure 4) compared with the neat baseline properties, with improvements of 11.37% and 10.71%, respectively.

### 3.3. Neat Vitrimer Flexion

**Figure 5:**
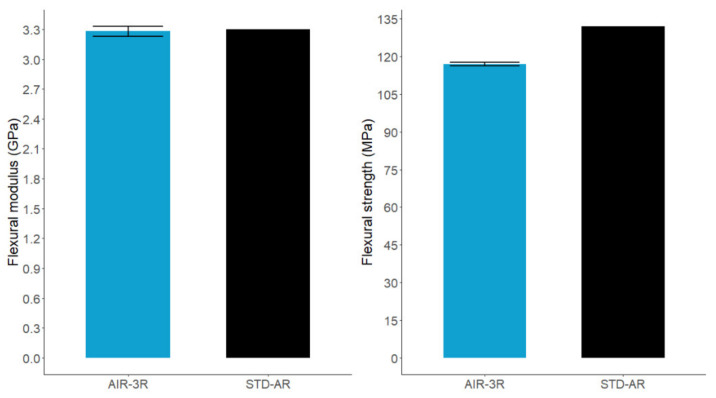
Flexural modulus (**left**) and strength (**right**) for the neat resin vitrimer and baseline reference.

The flexural modulus of the AIR-3R formulation was similar to the baseline, although the flexural strength was slightly lower, with a difference of −11.3%. It still had comparable mechanical behavior (Figure 5).

Other reported vitrimer formulations have quite similar tensile properties, most being slightly under of this formulation [9,10,13,20,23,71]. Only the Imine–Amine-based vitrimer formulation from Liu, H. et al. [2] and the 2,2-bis[4-(4-aminophenoxy)phenyl]propane formulation from Yuan, Y. et al. [6] presented better tensile properties related to the high rigidity of their polymer networks, the latter also having better flexural properties. Aranberri, I. et al. reported similar flexural properties on a DGEBA-disulfide vitrimer formulation [72]. The overall mechanical properties of the AIR-3R formulation are quite similar to the thermoset baseline, meaning that the use of this dynamic formulation would not affect the performance of structural components.

### 3.4. Tension

**Figure 6:**
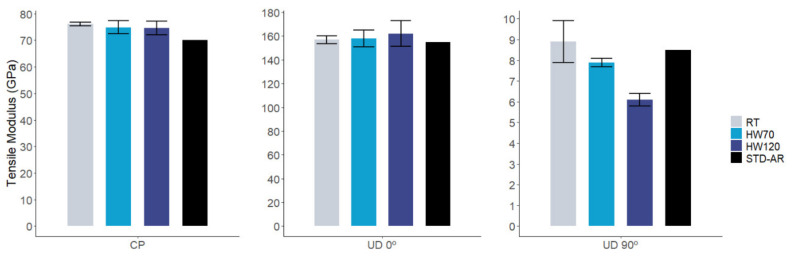
Comparative results in tensile modulus for AIR-3R vitrimer for different fabrics and test conditions. STD-AR composites tested under the three temperature and humidity conditions; one bar displayed as the final value was equivalent for the three cases (fiber dominant property).

**Figure 7:**
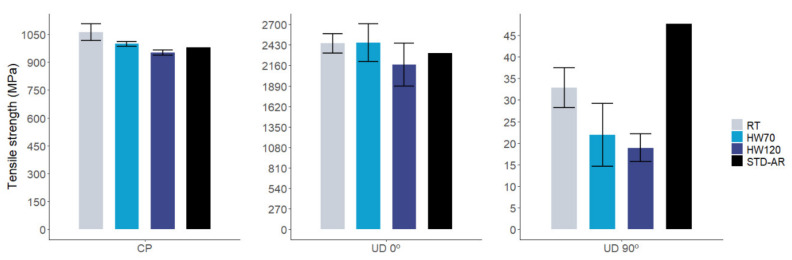
Comparative results in tensile strength for AIR-3R vitrimer for different fabrics and test conditions. STD-AR composites tested under the three temperature and humidity conditions; one bar displayed as the final value was equivalent for the three cases (fiber dominant property).

Figure 6 and Figure 7 show a comparison of the tensile modulus and tensile strength for the reinforcements studied under the different test conditions. For cross-ply and UD 0° reinforcements, tensile modulus performed slightly better in the three different conditions than the defined properties based on the aeronautic thermoset baseline: 8.7% and 1.3% at RT, 7% and 1.9% at HW70 and 6.6% and 11.9% at HW120, respectively. The same tendency is observed for tensile strength, with increments of 8.2% for cross-ply and 5.4% for UD 0° at RT and 1.8% (cross-ply) and 5.8% (UD 0°) for HW70. In the highest-temperature condition (HW120), AIR-3R performed slightly worse: −2.9% (cross-ply) and −6.7% (UD 0°). The T_g_ of the AIR-3R after aging was already lower than the pristine value, being in this case closer to the HW120 condition and possibly reducing its performance. However, we consider that under tension, most of the load is sustained by the reinforcement rather than the matrix. These results demonstrate that this new vitrimeric formulation does not interfere with the composite’s performance [9].

However, the UD 90° fabric’s performance was different: the relative variation of the modulus was 4.7%, −7% and −28.2% for RT, HW70 and HW120 conditions, respectively. The tensile strength decreased by −31.1%, −54% and −60.3% at RT, HW70 and HW120, respectively. In this matrix-dominated load case, the drop in mechanical performance is important if compared with the defined baseline. Hamada, H. et al. [73], and later Maurin, R. et al. [74], reported that tensile properties on UD CF in 90° are complex to determine. Fiber presence acts as a stress concentrator inside the matrix, so the matrix is a dominant factor, but the strength also depends on the fibers’ nature, chemistry and their interfacial properties and quality. Effects of the decreased T_g_ caused by the water uptake also have to be considered. Fracture analysis in the samples would be necessary in order to understand the dominant effect on the AIR-3R transverse tensile properties.

### 3.5. Compression

Compression test results present a high variability, inherent to the test setup. About 50% of the samples tested had to be discarded because of invalid failure modes. A percent bending strain (PBS) threshold of 10% was established for the test to be considered valid. Above this value, the coupon experiences non-neglectable load models such as bending, buckling or torsion.

**Figure 8:**
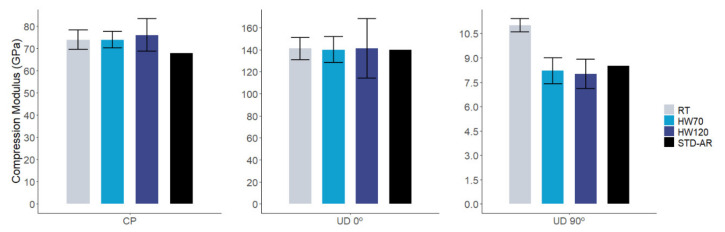
Comparative results in compression modulus for AIR-3R vitrimer for different fabrics and test conditions. STD-AR composites tested under the three temperature and humidity conditions; one bar displayed as the final value was equivalent for the three cases (fiber dominant property).

Compression modulus (Figure 8) of cross-ply fabric composites with AIR-3R vitrimer was higher than the defined baseline by 8.8% in RT and HW70 and 11.9% in HW120. For the UD 0° composite with the vitrimer, the compression modulus was within 1% above the reference. In the UD 90° fabric, at RT, AIR-3R overpassed the thermoset formulation, but under high temperature and humidity, it presented a slightly inferior response, albeit still comparable with the thermoset counterpart: 22.2% in RT, −3.5% in HW70 and −5.9% in HW120.

**Figure 9:**
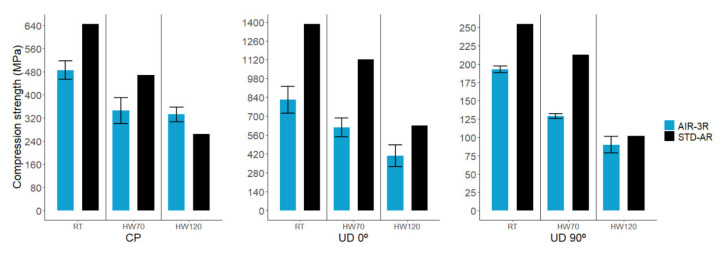
Comparative results in compression strength for AIR-3R vitrimer for different fabrics and test conditions. Samples with the PA veil.

Compression strength (Figure 9) of the vitrimer composites presented an abrupt drop in most of the conditions: −24.8% in RT, −26.3% in HW70 and 25.6% in HW120 for the cross-ply fabric; −40.6% in RT, −44.9% in HW70 and −35.3% in HW120 for UD 0°; and −24.2% in RT, −39.3% in HW70 and −11.8% in HW120 for UD 90°.

**Figure 10:**
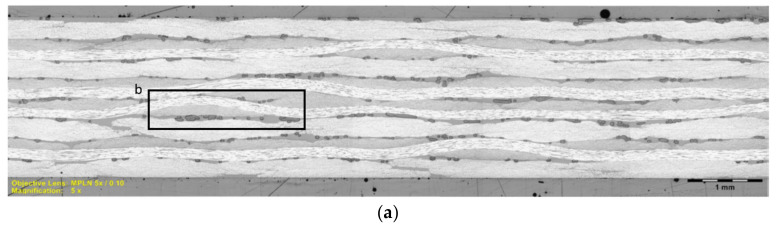

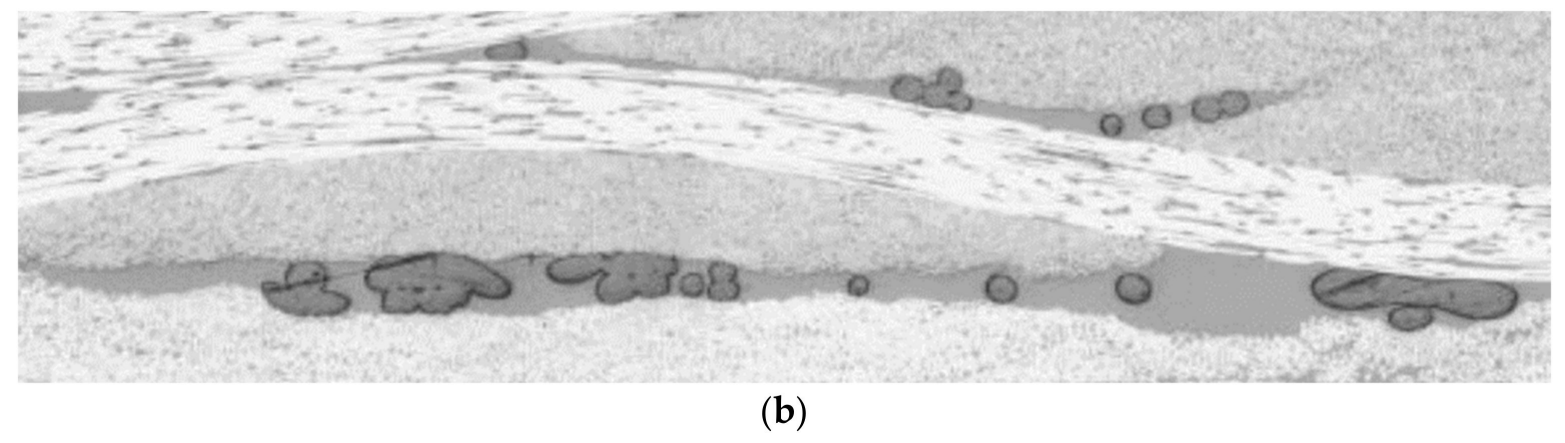
Micrography images of AIR-3R composite sample. (**a**) Panel cross-section. (**b**) Detail of layers interface in the laminate. Objective lens MPLN 5X/0.10.

To better understand this compressive strength behavior, we took micrography images (Figure 10) of the AIR-3R samples. Some gray-rounded areas appear between the fiber layers corresponding to the thermoplastic PA veil initially included in the carbon fiber fabrics. According to the manufacturer, this veil has a melting temperature close to 180 °C that corresponds to the in-mold post-curing temperature of the AIR-3R vitrimer formulation. In the first curing step (130 °C), the vitrimer starts to polymerize, embedding the veil structure inside the laminate. Therefore, even if this veil later melts, it is trapped by the already frozen structure of the vitrimer matrix, possibly acting as a contaminant or stress concentrator.

**Figure 11:**
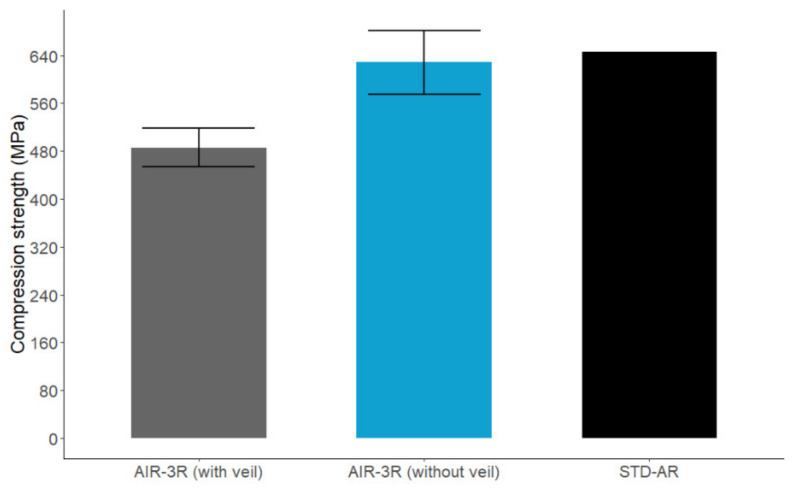
Comparison of the compressive strength for the AIR-3R vitrimer with and without the PA veil and the base thermoset reference. Cross-ply fabric tested under RT condition.

The PA veil was included in the AIR-3R vitrimer composite in order to enhance the interlaminar toughness and impact resistance, as has been demonstrated in several studies addressing interleaving thermoplastic veils [75,76,77,78,79,80,81,82,83,84]. In view of this microstructure, a new batch of tests was performed on samples without the thermoplastic PA veil; i.e., it was removed and all other conditions were maintained. The AIR-3R vitrimer performed comparably to the thermoset baseline reference with a slight difference of −2.8% in the compression strength. The presence of the PA veil caused a decrement in the compressive response of the vitrimer composite (Figure 11). Related publications detail that thermoplastic veils acting as interleaves often increase the fracture toughness by serving as an obstacle to crack propagation but, as a countereffect, lowering the composite’s in-plane properties. Thermoplastic veils that do not melt during the manufacturing process have more permeability than the fibers, thus creating resin-rich zones and promoting voids. The higher the veil’s aerial weight, the more the detrimental effect on the mechanical response is accentuated [77,78,80,85,86,87]. One of these studies reports that with a 4 g/m^2^ co-PA veil, the compression strength decreases by 9%. In this work, the PA veil had double the aerial weight, which negatively impacted the compression strength by 22.6%.

In view of the results with and without veil, it is concluded that the AIR-3R vitrimer formulation has no detrimental effect on the compressive strength of the final composite, thus being mechanically comparable to the thermoset baseline resin.

When comparing it to the vitrimers reported in the literature, AIR-3R has a superior compressive strength in reference to the 2,2-bis[4-(4-aminophenoxy)phenyl]propane-based formulation from Yuan, Y. et al. [6]. In this point, we have to highlight that their neat vitrimer properties were higher that the AIR-3R formulation. The difference could be related to the lower fiber content in their composites (50.1% for a plane-weave fabric in a cross-ply configuration). It is also superior in compressive strength to the vitrimer from Ruiz de Luzuriaga, A. et al. [9], which has a glass fiber reinforcement instead of carbon fiber.

### 3.6. Interlaminar Shear Strength

**Figure 12:**
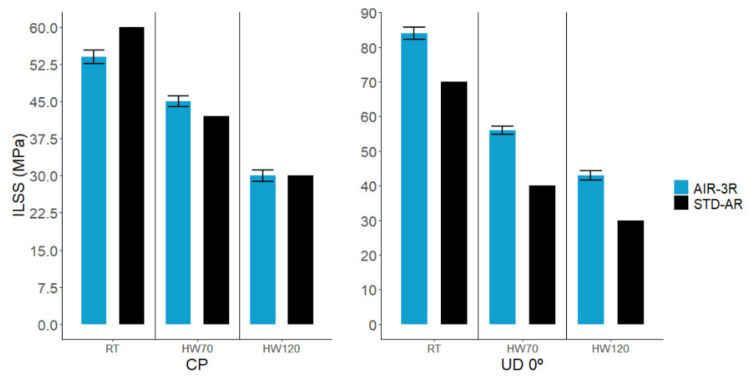
ILSS shear performance of the AIR-3R vitrimer formulation for different fabrics and test conditions.

In all temperature conditions, the AIR-3R vitrimer performed well (Figure 12). The cross-ply fabric response was comparable to the baseline thermoset. Only at RT did it slightly underperform: −10% in RT, 7.1% in HW70 and 0% in HW120. For the UD, shear performance was superior to the baseline, even reaching an outstanding performance at the higher temperature condition: 20% in RT, 40% in HW70 and 43.3% in HW120.

**Figure 13:**
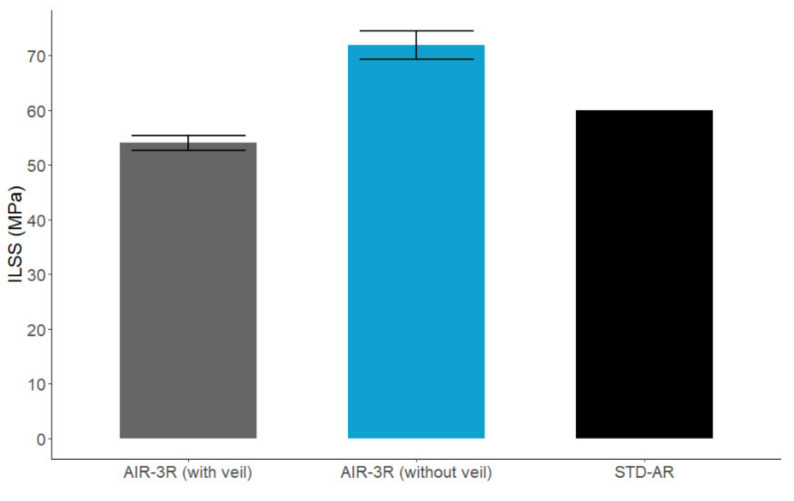
Comparison of the interlaminar shear strength for the AIR-3R vitrimer with and without the PA veil and base thermoset reference. Cross-ply fabric tested under RT condition.

In view of the influence of the PA veil on the compression response, another batch of ILSS samples was manufactured with the cross-ply fabric without the PA veil and tested at RT. These samples showed better performance than the original testing set, surpassing the baseline thermoset by 20% (Figure 13) and demonstrating than the presence of the PA veil in the laminate causes a 24.89% loss in the interlaminar shear performance.

### 3.7. In-Plane Shear

**Figure 14:**
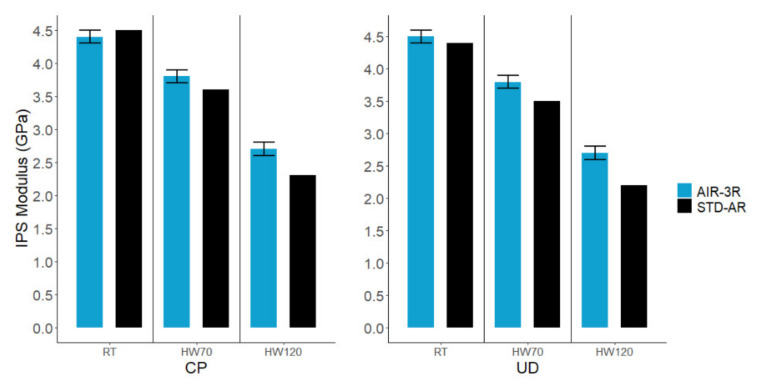
In-plane shear modulus of the AIR-3R vitrimer formulation for different fabrics and test conditions.

The IPS modulus for the vitrimer composite was generally better than the thermoset-based composite in both cross-ply and UD fabrics, particularly in high-temperature conditions (Figure 14): −2.2% in RT, 5.5% in HW70 and 17.4% in HW120 for CP fabric, and 2.3% in RT, 8.6% in HW70 and 22.7% in HW120 for UD.

**Figure 15:**
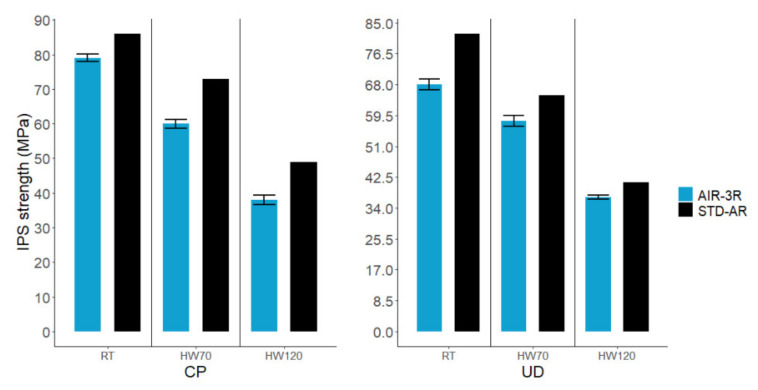
In-plane shear strength of the AIR-3R vitrimer formulation for different fabrics and test conditions.

Regarding the in-plane shear strength (Figure 15), the vitrimer composite underperformed the baseline: −10% in RT, −17.7% in HW70 and −21.5% in HW120 for the cross-ply fabric, and −17.1% in RT, −11.5% in HW70 and −10.5% in HW120 for the UD.

The ILSS shear strength and IPS shear modulus suggest that the AIR-3R vitrimer has good toughness, good adhesion to the reinforcements, good adhesion between layers and good shear resistance, even after hot wet conditioning of the specimens. As was seen for the ILLS, the lower in-plane shear strength could be attributed to the thermoplastic PA veil inside the laminate.

AIR-3R ILSS performance was slightly higher compared with other vitrimer formulations, being comparable with the Imine VA/HTDA system [20]. The BAPP vitrimer system [6] has better shear performance, due to the network structure. The Vurea–Amine vitrimers [25,26] had comparable IPS shear modulus (4.7 GPa) to the AIR-3R vitrimer composite but lower shear strength (41 MPa).

### 3.8. Open-Hole Tension, Compression and Filled-Hole Compression

#### 3.8.1. Open-Hole Tension

**Figure 16:**
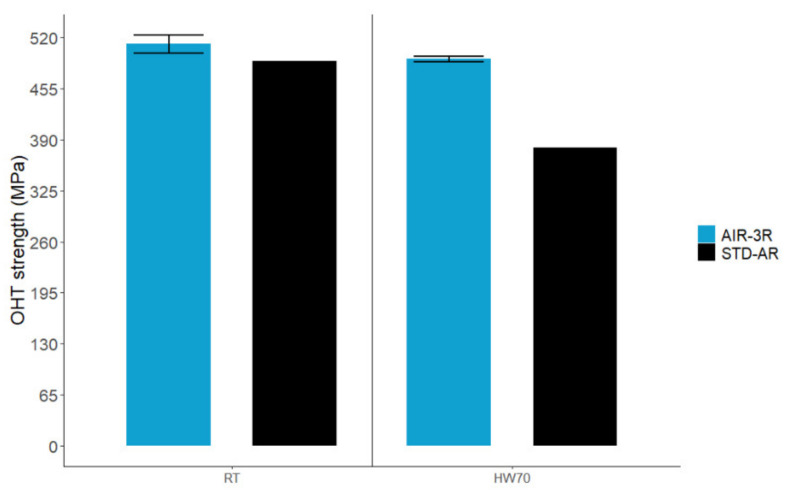
OHT test for the AIR-3R vitrimer composite under test conditions.

**Figure 17:**
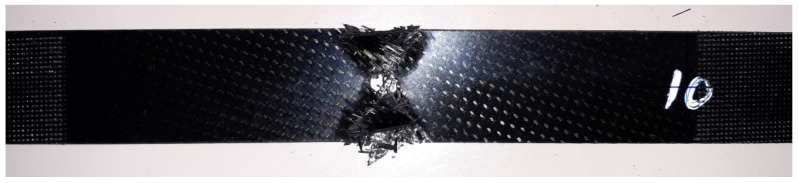
AIR-3R failed specimen under OHT test.

At RT conditions, the AIR-3R formulation is comparable to the base thermoset resin (Figure 16). After moisture saturation, testing at 70 °C presented a superior strength: 4.5% and 29.8%, respectively. All specimens presented good failure modes (Figure 17). Again, fibers carry most of the load, indicating no alternations in composite behavior.

#### 3.8.2. Open-Hole Compression

**Figure 18:**
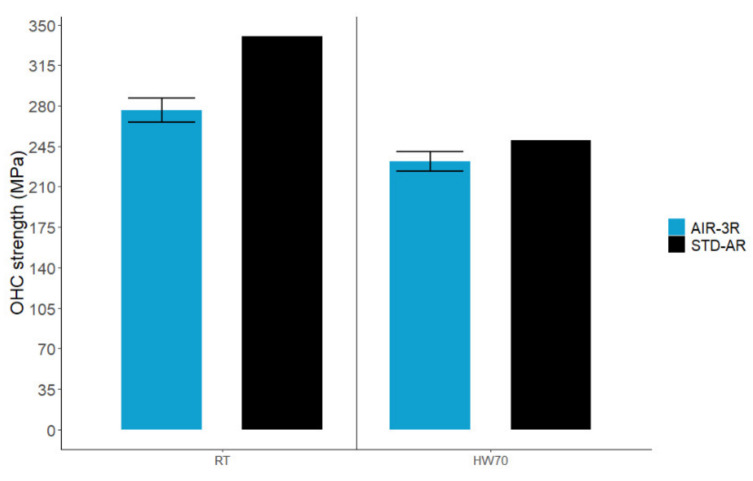
OHC test for the AIR-3R vitrimer composite under test conditions.

Specimens had a proper failure mode under the specified standard. The AIR-3R vitrimer had a slightly lower compressive strength than the thermoset baseline (Figure 18). The PA veil is likely behind these results, considering the effect on the compression and shear tests.

#### 3.8.3. Filled-Hole Compression

**Figure 19:**
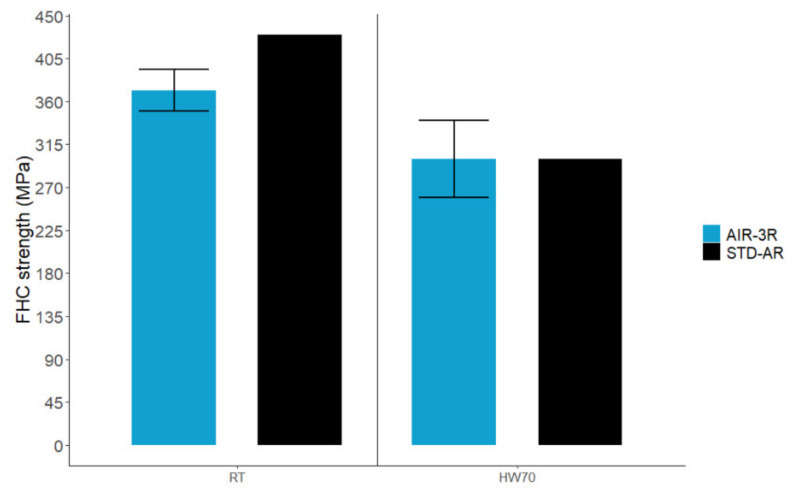
FHC test for the AIR-3R vitrimer composite under test conditions.

The FHC strength for the AIR-3R vitrimer composite was slightly lower (−13.6%) than the baseline at RT and reached the same value for the HW70 condition (Figure 19). All the specimens presented non-valid failure modes, as they failed outside the bolt area. The attempts to improve this issue by tightening the jig bolts and by ensuring a correct alignment of the sample and parallelism of the loading plates did not succeed.

Nevertheless, the results are still comparable to the base thermoset composite. There are no reports of other vitrimer formulations having been tested on OHT, OHC and FHC.

### 3.9. Interlaminar Fracture Toughness

**Figure 20:**
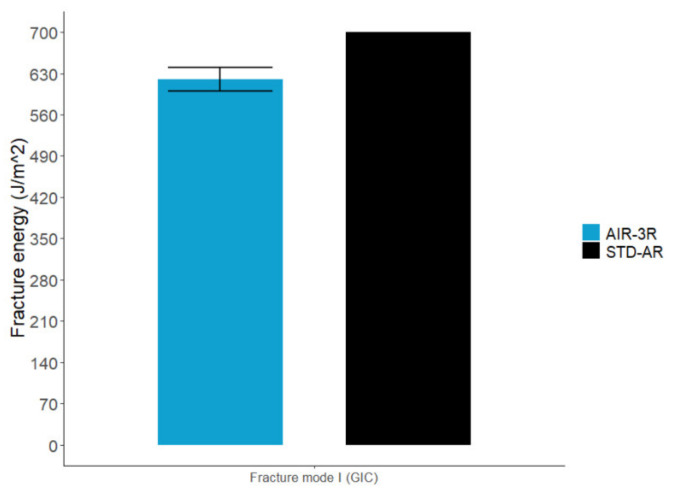
Fracture toughness for AIR-3R vitrimer composite.

The AIR-3R vitrimer specimens presented lower fracture toughness under mode I (G_IC_) at RT but were still comparable to the base thermoset resin in that they exhibited a 11.4% difference (Figure 20).

A baseline value for the fracture toughness under mode II (G_IIC_) was not defined. The AIR-3R vitrimer composite presented a mean energy value of 876.19 J/m^2^.

Fracture toughness under mode I (G_IC_) was comparable to reported thermoset composites toughened with thermoplastic veils: T300 UD CF 167 g/m^2^–Epoxy RTM6-2 with 20 wt% PAEK veil [88], UD CF 350 g/m^2^–Epoxy L160 with 17 g/m^2^ PA-66 veil [75], Epoxy MTM57/T700S (24K) UD prepreg with 4.5 g/m^2^ and 9 g/m^2^ PA-66 veils [89]. Despite the differences in the veil materials, toughening mechanisms and mechanical responses were similar. Factors such as veil polymer type and aerial weight were more important in the performance of the final laminate.

Mode II (G_IIC_) fracture toughness was lower than in similar thermoset composites toughened with thermoplastic veils [79,88,89,90]. Toughening mechanisms in mode II (G_IIC_) were more complex than mode I (G_IC_), depending on most on the neat properties of the matrix and the architecture of the reinforcement, rather than the fiber bridging effect. The clarification of this topic deserves further research.

## 4. Conclusions and Outlook

The neat vitrimer properties demonstrate that this formulation can be used as a matrix for high-performance structural components, having similar properties to the thermoset baseline and thus not affecting their performance. This new epoxy–disulfide vitrimer composite has good in-plane stiffness under tension and compression and good shear stiffness, denoting good adhesion between fibers at the interface. Vitrimer composite strength under compression and interlaminar shear was proven to be highly influenced by the presence of the un-melted thermoplastic veil, which had been intended to enhance the fracture toughness. Micrography analysis and comparison to related studies show that the un-melted veil creates brittle resin-rich zones. Compression samples without the PA veil demonstrated that this trapped interface in the laminate reduces the compressive strength by 22.6%, while the ILSS samples presented reductions of 24.89%. The interlaminar veils could also have influenced the tension strength at UD 90°, in-plane shear strength, OHC and FHC strength, all of which were lower than the base thermoset formulation.

Fracture toughness in mode I (G_IC_) was comparable to reported thermoset formulations with toughening thermoplastic veils. Mode II appears to be lower than the references with and without the thermoplastic veils. Further research should be carried out to clarify the micro-mechanisms behind the fracture behavior of this vitrimer formulation, as well as the impact of the thermoplastic veils, their melting temperature and aerial weight on the in-plane vitrimer composite properties.

In summary, the mechanical properties of the AIR-3R and the composites prepared with this new vitrimer formulation were comparable to those currently used in aircraft materials. Dynamic properties of this particular formulation have to be studied in order to establish a processing window in which this formulation could be reprocessed by maintaining the overall composite properties.

## Figures and Tables

**Table 1:** Summary of reported properties for vitrimer composites and some thermoset aeronautic-grade references.

Reference	Fiber/Fabric	Resin/Monomer	Dynamic System/Hardener	Tensile Modulus [GPa]	Tensile Strength [MPa]	Compression Strength [MPa]	Flexural Modulus [GPa]	Flexural Strength [MPa]	ILSS [MPa]	Impact Strength [kJ/m^2^]	Tg [°C]
Ruiz, A. et al. [9]	Glass	Diglycidyl ether of bisphenol A	4-aminophenyl disulfide	-	-	292 ± 16	-	595 ± 39	37 ± 3	194 ± 18	130
Denissen, W. et al. [25,26]	Glass—plain weave (hot-pressed)	Vinylogous urethane	Amine	33.2	336	-	-	-	-	-	-
Liu, X. et al. [32]	Glass cloth (180 g/m^2^)	Phenol formaldehyde	Urethane	-	-	-	-	184.1	12.93	-	157
Si, H. et al. [10]	Carbon	Bis(4-glycidyloxyphenyl)disulfide	4-aminophenyl disul-fide	10.5	334.5 ± 87.7	-	-	-	-	-	147
Yuan, Y. et al. [6]	Carbon woven T300-1000 (119 g/m^2^)	Poly(hexahydrotriazine)	2,2-bis[4-(4-aminophenoxy)phenyl]propane	68.3	741.2	351.5	54.8	829.7	75.5	-	198.2
	Carbon UD HS (200 g/m^2^)			141.7	1806.6	343.3	127.4	1241.2	69.1	-	199.5
Taynton, P. et al. [28]	Carbon—twill weave	Diamine	Polyimine	14.2 ± 1.1	399 ± 85	-	32.4 ± 3.7	255 ± 56	-	-	145
Wang, S. et al. [31]	Carbon	Phenol formaldehyde	Boronic ester	-	-	-	24.2 ± 0.3	411.6 ± 5.3	48 ± 2.5	-	153
Liu, Y. et al. [19]	Carbon	Itaconic acid-based epoxy	Maleic acid + glycerin	31.3	417	-	-	-	45	-	54
Wang, S. et al. [23]	Carbon—plain weave	Vanillin epoxy	Diamine 4,4′-methyl- enebiscyclohexanamine	35.3 ± 2.4	763 ± 71	-	-	-	-	-	172
Liu, Y.Y. et al. [35]	Carbon	Bio epoxidized soybean oil	Vanillin + 4-aminophenol	1.18 ± 0.14	145.4 ± 17.13	-	-	-	-	-	27.6
Liu, Y.Y. et al. [36]	Carbon—plain weave	Glycerol triglycidyl ether	Vanillin + A4-aminophenol	12.9	449	-	-	-	-	-	70
Memom, H. et al. [20]	Carbon	Trifunctional epoxy	Vanillin + methylcyclohexanediamine	-	-	-	56	1028	52	-	131
Liu, T. et al. [33]	Carbon	Bisphenol A + Ethylenediamine	Glutaric anhydride	17.1 ± 2.5	356 ± 28.7	-	-	-	-	-	95
Wang, H. et al. [34]	Carbon (low wt%)	Diglycidyl ether of bisphenol F	4-Aminophenyl disulfide + γ-Aminopropyltriethoxysilane + Poly(propylene glycol) bis(2-aminopropyl ether)	10.18	320	-	-	-	-	-	97.4
Xu, Y. et al. [37]	Carbon (braided 60% FVF)	Epoxidized menthane dia- mine	Adipic acid	-	465	-	-	-	-	-	86.4
Aeronautic standard reference [6]	Carbon–5 h satin (prepreg)	Hexply 914	-	70	631	-	61	912	64	-	190
Aeronautic standard reference [38]	Carbon 8H satin 6K (prepreg)	Hexply 8552	-	86	1014	-	-	-	90	-	200
Aeronautic standard reference [39]	Carbon UD (prepreg)	Epikote 475	-	-	-	-	60	1020	65	-	190
Aeronautic standard reference (approximated results) [40]	Carbon–5H satin (370 g/m^2^)	RTM6	-	69	1180	950	-	-	67.5	-	-

**Table 2:** Baseline specifications for the AIR-3R resin based on conventional STD-AR. Adapted from [47].

**Material**	**Specification**	**Unit**	**Value**					
Neat resin	Glass transition temperature	°C	>170					
	Tensile modulus	GPa	3					
	Tensile strength	MPa	75					
	Flexural modulus	GPa	3.3					
	Flexural strength	MPa	132					
**Material**	**Specification**	**Unit**	**Fabric configuration and test conditioning**					
			**CP**			**UD**		
			**RT**	**HW70**	**HW120**	**RT**	**HW70**	**HW120**
Ply properties	Tensile modulus—warp direction	GPa	>70			>155		
	Tensile modulus—weft direction	GPa	>70			>8.5		
	Tensile strength—warp direction	MPa	>980			>2325		
	Tensile strength—weft direction	MPa	>980			>47.6		
	Compression modulus—warp direction	GPa	>68			>140		
	Compression modulus—weft direction	GPa	>68			>8.5		
	Compression strength—warp direction	MPa	>646			>1386		
	Compression strength—weft direction	MPa	>646			>255		
	Interlaminar shear strength	MPa	>60	>42	>30	>70	>42	>30
	In-plane shear modulus	GPa	>4.5	>3.6	>2.3	>4.4	>3.5	>2.2
	In-plane shear strength	MPa	>86	>73	>49	>82	>65	>41
**Material**	**Specification**	**Unit**	**Quasi-isotropic laminate**					
			**RT**		**HW70**		**HW120**	
Laminate properties	Fracture toughness G_IC_ *	J/m^2^	>700					
	OHT strength	MPa	>490		>380		>360	
	OHC strength	MPa	>340		>250		>180	
	FCH strength	MPa	>430		>300		>275	

* Fracture toughness mode II (G_IIC_) is not available on the baseline data.

## Data Availability

Not applicable.
